# Global interlaboratory study: Assessing consistency and accuracy for determining source of face, hands, and clothing images across forensic practitioners and laypersons

**DOI:** 10.1111/1556-4029.70368

**Published:** 2026-06-15

**Authors:** Kelly A. Meiklejohn, Chau‐Wai Wong, Melissa K. R. Scheible, Yang Qu, Jane Harris, Lora Sims

**Affiliations:** ^1^ Department of Population Health and Pathobiology North Carolina State University, College of Veterinary Medicine Raleigh North Carolina USA; ^2^ Department of Electrical and Computer Engineering North Carolina State University, College of Engineering Raleigh North Carolina USA; ^3^ Clinical Studies Core, Office of Research North Carolina State University, College of Veterinary Medicine Raleigh North Carolina USA; ^4^ Center for Forensic Science Northern Michigan University Marquette Michigan USA; ^5^ Face Center of Excellence (FaCE) Ideal Innovations, Inc. Arlington Virginia USA

**Keywords:** clothing comparison, digital multimedia, facial comparison, hand comparison, image comparisons, opinion categories, OSAC proposed standard

## Abstract

Images containing faces, body parts, vehicles, weapons, clothing, luggage, furniture, landscapes, and buildings are routinely sent to forensic laboratories for comparison. Trained forensic practitioners offer an opinion regarding a common source or a different source. Results from comparisons are typically reported using opinion categories, and the number used can vary (typically 3–7 categories). To harmonize opinion categories used across digital multimedia disciplines, the Organization of Scientific Area Committees (OSAC) for Forensic Science developed OSAC Proposed Standard *2022‐S‐0001 (Standard Guide for Image Comparison Opinions*), which details five opinion categories for determining image source. To ensure this standard meets the OSAC program goal of developing technically sound standards, an interlaboratory study focused on *2022‐S‐0001* was conducted. Combining machine learning and input from subject matter experts, 20 pairs of face, hands, and clothing images were selected for use that represented varying sources and difficulty. Qualtrics was used to disseminate a survey and collect responses from forensic practitioners (*n* = 38) and laypersons (*n* = 44). For all three image subject categories, statistically higher agreement (selection of the same opinion source category for the same pair of images) was observed between practitioners over laypersons. Practitioners were statistically more accurate at determining the true source over laypersons for face (*p* = 0.005) and hand (*p* = 0.043) image pairs. This study highlighted that when individuals are provided with a clear and detailed framework of source opinion categories, moderate agreement and high accuracy (especially for practitioners) can be achieved for face, hand, and clothing image comparisons.

**Highlights**

- OSAC proposed standard *2022‐S‐0001* details five opinion categories for determining the image source.
- Forensic practitioners and laypersons were recruited to assess the technical soundness of *2022‐S‐0001*.
- Participants were given face, hand, or clothing images and determined the source using *2022‐S‐0001*.
- Practitioners were statistically more accurate at determining the true source of face and hands images over laypersons.
- Practitioners had statistically higher agreement than laypersons for face, hand, and clothing images.

## 1 INTRODUCTION

Photographs and videos that capture an individual, subject, or scene have become almost synonymous with every aspect of our lives, given the ubiquitous access to cameras in cell phones. It has been estimated that every day 93 million selfies are taken globally [1], and nine out of ten Americans own a smartphone capable of capturing images and videos [2]. In addition to photos and videos taken by cell phones, the estimated 1 billion closed‐circuit TV (CCTV) cameras globally are constantly recording [3]. Given the prevalence and access of photos and videos in society, their use as evidence in forensic investigations will likely only increase in the coming decades.

Comparison of images for forensic purposes is defined by the Scientific Working Group on Digital Evidence (SWGDE) as “the assessment of the correspondence between features in questioned items depicted in images and either questioned or known objects or images, for the purpose of rendering an expert opinion regarding identification or elimination” [4]. Virtually any item or subject captured in an image can be subjected to forensic examination, including but not limited to faces (and other body parts/areas), vehicles, weapons, clothing, luggage, furniture, landscapes, and buildings [4]. Forensic image comparisons are frequently referred to as “side‐by‐side” comparisons since they usually involve a comparison of class (e.g., vehicle make, model, year) and individualizing characteristics (e.g., broken taillight, dent in bumper) in imagery [5]. There are four main application categories of forensic image comparisons: (1) intelligence gathering, compilation of information and images believed to be of the single subject/location even if the identity of the subject/location is not known, (2) screening and access control, which involves either image‐to‐image or image‐to‐person comparisons commonly implemented at customs and immigration checkpoints, (3) investigative and operational leads, comparisons not intended for court presentation but rather to narrow down a list of potential subjects/locations for investigators, and (4) forensic comparisons, presented as evidence to either a judge or jury [6].

During a side‐by‐side comparison of images, practitioners are tasked with examining similarities and differences to determine whether they depict the same source as opposed to the hypothesis that they depict a different source. Opinion categories (also referred to as opinion scales) are subsequently used to describe the relative level of support provided by the data. Various scales exist, with the most common being 3‐point, 5‐point, and 7‐point scales, and the scale used can vary depending on the application (e.g., when the result of an image comparison is reported as an investigative lead, as opposed to a full forensic analysis) and what is specified by laboratory procedures.

In recent years, several scientific studies have been conducted to assess the accuracy and reproducibility of forensic image comparisons completed by trained practitioners. Notably, the majority of these have focused on facial image comparisons (e.g., [7, 8, 9]) and have included performance assessments of trained forensic practitioners, computer algorithms, and laypersons. A smaller number of studies have focused on image comparisons of clothing [10], hands [11, 12], and vehicles [13]. Overall, the consensus from most of these studies is that trained forensic practitioners are highly accurate and outperform computer algorithms and laypersons with difficult image comparisons.

To harmonize opinion categories used across practitioners, a task group from the Organization of Scientific Area Committees (OSAC) for Forensic Science worked to develop OSAC Proposed Standard *2022‐S‐0001*: *Standard Guide for Image Comparison Opinions* [14]. This proposed standard provides a 5‐point scale for opinions that can be reached by a practitioner performing comparisons of people, objects, or scenes captured in images: 5.1—strong support for different sources, 5.2—support for different sources, 5.3—inconclusive, 5.4—support for a common source, and 5.5—strong support for a common source. Given (a) the diversity of image subjects that this OSAC proposed standard encompasses, and (b) that a goal of OSAC is to develop and promote the use of high‐quality, technically sound standards, it was prudent to conduct an interlaboratory study to assess the practical utility, accuracy, and reproducibility of OSAC Proposed Standard *2022‐S‐0001*. To achieve this, we recruited two cohorts of participants—forensic practitioners deemed competent in image comparisons and laypersons—and asked them to select an image source opinion from *2022‐S‐0001* for a consistent set of either 20 face, hand, or clothing image pairs.

## 2 MATERIALS AND METHODS

**FIGURE 1:**
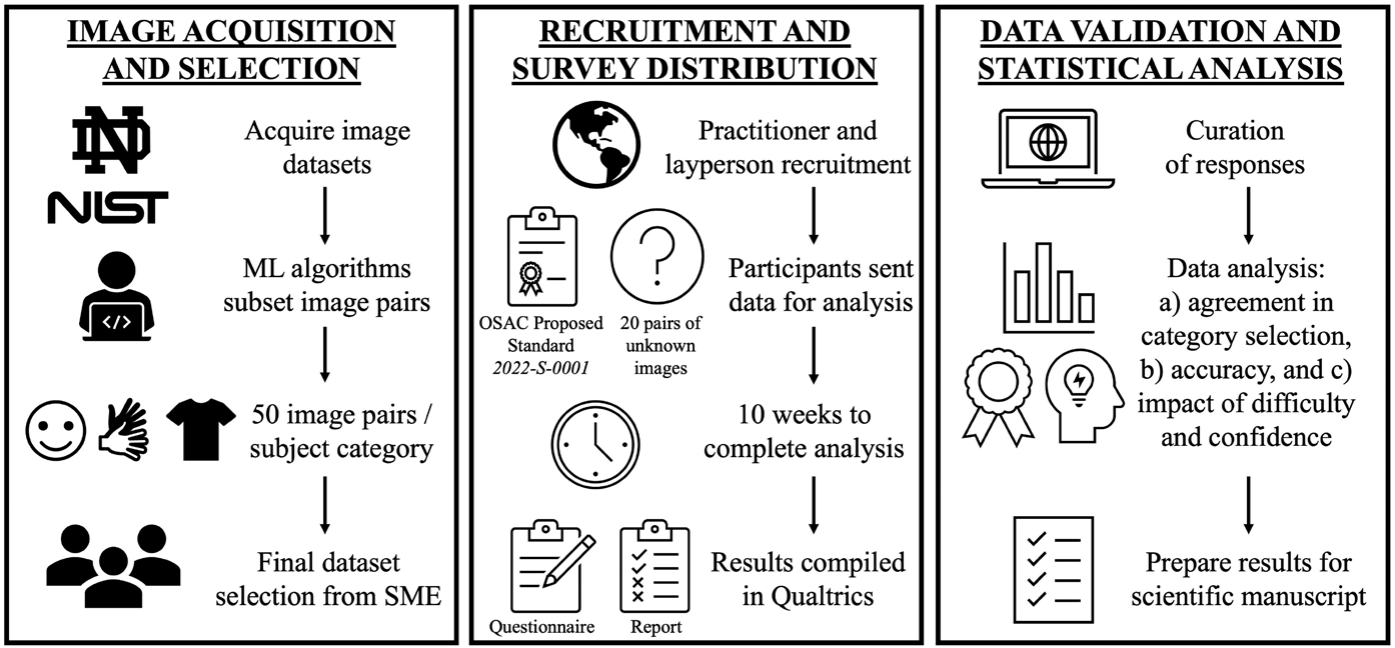
Schematic for the interlaboratory study, which outlines key steps within each phase of the project. ML, machine learning; SME, subject matter expert.

The overall experimental design for this global interlaboratory study is given in Figure 1.

### 2.1 Image acquisition and selection

The scope of *2022‐S‐0001* states that this proposed standard only provides a framework for “performing comparisons of people, objects, or scenes captured in images” and “is not intended for the comparison of images of impressions (e.g., tool marks, friction ridge).” Given this, the image subjects chosen for this interlaboratory study were limited to only still images (i.e., not videos) for three categories: faces, hands, and clothing. The rationale for this choice was twofold: (1) these subject categories represent comparisons frequently completed in casework, meaning a larger number of forensic practitioners would be eligible to participate in this interlaboratory study and (2) statistical analyses will have greater power, given a higher number of suitable practitioner participants could be recruited (i.e., if more obscure image subject categories were included, it would be difficult to find a statistically sufficient number of forensic practitioners to complete the comparisons).

Existing image datasets maintained by the University of Notre Dame Computer Vision Research Laboratory and the National Institute of Standards and Technology (NIST) were licensed to North Carolina State University for use in this study (see Supporting Information 1 for the list of datasets). A total of 125,000 images were accessed across 12 datasets. Machine learning (ML) algorithms (see Supporting Information 2 for technical details) were used to prefilter images, narrowing them down to 100 pairs of face images, 100 pairs of hand images, and 45 pairs of clothing images. A pair could come from the same person/item (i.e., common source) or two different people/items (i.e., different sources). For the face and hand categories, the ML algorithms additionally selected pairs that could be easily or difficultly differentiated by well‐established computer vision criteria. Images not selected included those that (a) had been manipulated (e.g., retouched, saved in reduced pixel format), (b) represented varied quality/size, and (c) for facial images, were not frontal views. For the facial image sets, considerations were made to ensure that image pairs were as demographically varied as possible with regard to general physical appearance, individual age, and gender. For the clothing image pairs, only clothing from the torso (*n* = 10) and neck and shoulders (*n* = 10) were available for use and consisted of either shirts (with and without collars), jackets, or hooded sweaters. Body shape was largely undiscernible in the clothing images available for examination. The majority of the clothing pairs consisted of plain clothing (i.e., solid colors; *n* = 13), as opposed to those with patterns (*n* = 4) or identifiable logos (*n* = 3). We automated open‐source algorithms for face and clothing selections and developed an in‐house algorithm for hand image selection due to the lack of available open‐source algorithms. The automation code, in‐house hand image selection algorithm, and detailed documentation are available at https://bitbucket.org/leecwwong/image‐comparison/.

**FIGURE 2:**
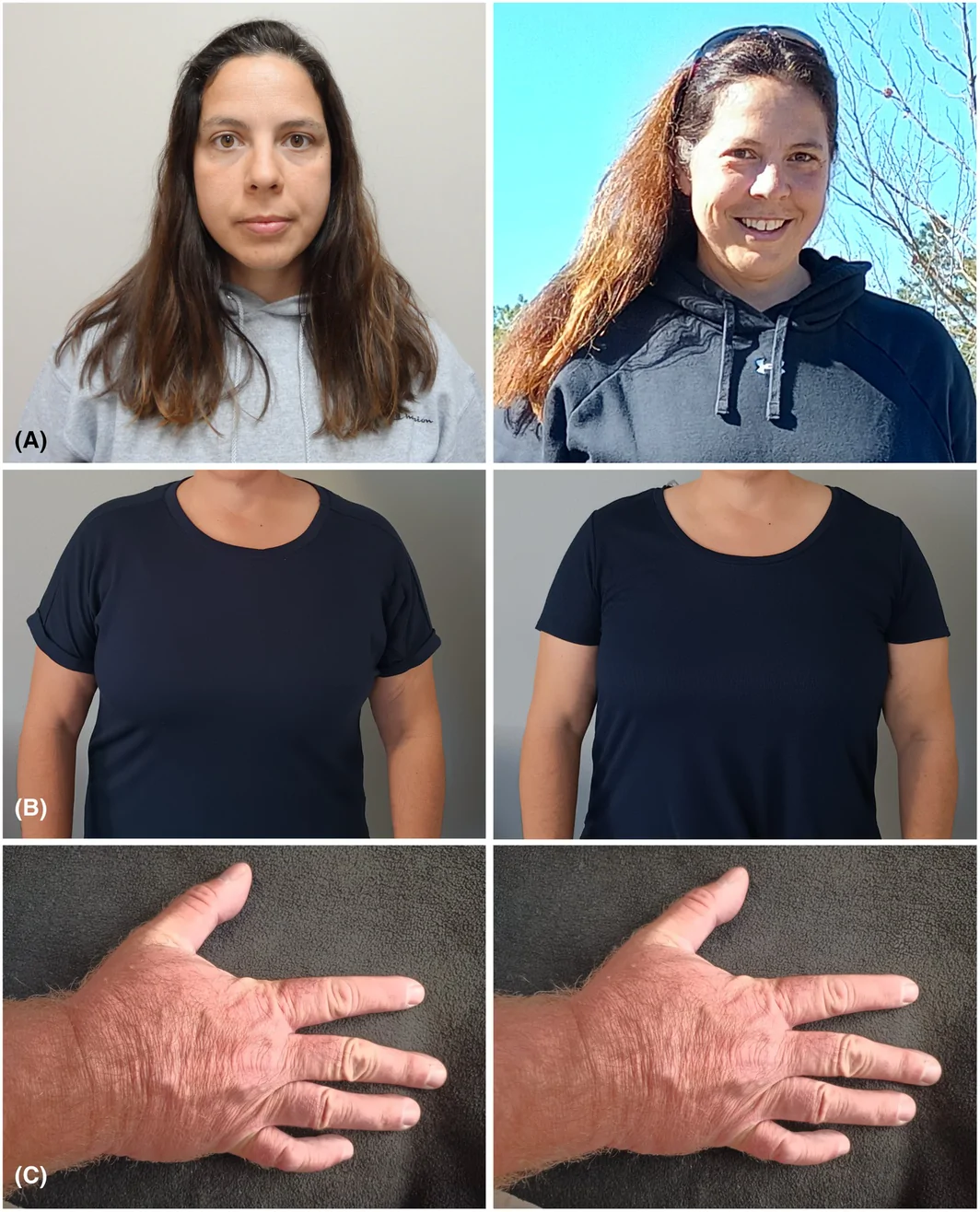
Example comparison of face (A), clothing (B), and hand (C) image pairs.

Subject matter experts—individuals deemed competent in face, hand, and/or clothing image comparisons and complete image comparisons for forensic applications—were tasked with selecting the final 20 image pairs for each of the three categories. They were instructed to select image pairs that spanned varying source (i.e., common, different) and difficulty (i.e., easy, difficult). The total number of pairs corresponding to source and difficulty (i.e., common source—difficult, common source—easy, different source—difficult, and different source—easy) was not evenly distributed within each image subject category. This imbalance was intentional to reduce the risk of participants deducing the source by process of elimination. The final list of 120 images selected for use in this study was all derived from the University of Notre Dame datasets (Supporting Information 1). To prepare these images for insertion into the Qualtrics survey (Version 2024; Seattle, WA, USA), two final steps were completed: (1) any head, neck, or body was cropped from clothing images to ensure comparisons were based only on the clothing itself and (2) zoom and size of face and clothing image pairs were made consistent to mimic casework and reduce any effects of having images at varied zoom on the source opinion selected. For example, inconclusive might have been chosen for a pair of face images that included (a) one close/zoomed in image alongside an image of an individual standing at a distance or (b) at least one image with obstructions, such as hair covering part of the face. An example of a typical face, hand, and clothing image pair for comparison is given in Figure 2; these images were not those provided to participants, and permission was given from individuals captured in these images for inclusion in this paper.

### 2.2 Participant recruitment and survey distribution

Two cohorts of individuals were recruited for this interlaboratory study: (1) Forensic practitioners, those deemed competent by their respective agency, organization, or other entity and currently conduct forensic casework on face, hand and/or clothing image comparisons and (2) Laypersons, those not trained in forensic image comparisons of face, hands, or clothing. The main approach to recruit participants from the practitioner cohort was through relevant organization or society mailing lists (e.g., Scientific Working Group on Digital Evidence [SWGDE], Facial Identification Scientific Working Group [FISWG], OSAC Facial Identification and VITAL subcommittee members and affiliates, American Academy of Forensic Science [AAFS] Digital and Multimedia newsletter) and advertisement at scientific conferences relevant to the digital multimedia community. Laypersons served as an important cohort for this study given (a) in the scope of *2022‐S‐0001* it was identified that limited work has been done to assess how laypersons interpret opinion categories and (b) laypersons are increasingly likely to be exposed to OSAC standards—including opinion scales like the one evaluated in this study—as they are adopted by more practitioners and could be referenced in court proceedings, so they need to be understandable by non‐experts. For the layperson cohort, we identified three subgroups of individuals who were recruited as follows (a) students involved in forensic science, recruited via mailing lists of universities with forensic science degree programs (Pennsylvania State University, Virginia Commonwealth University, Sam Houston State University, West Virginia University, North Michigan University), (b) individuals trained in forensic science but not image comparisons, recruited via the OSAC newsletter and a OSAC LinkedIn post, and (c) general public, recruited by co‐authors of this study posting on LinkedIn and sharing with mailing lists through their respective universities or facilities. A Google form link was shared in all recruitment efforts, which captured from interested participants: (a) contact information, (b) jurisdiction, country, and image category competency (practitioners only), and (c) experience and exposure to forensic science (laypersons only).

Based on responses to the Google form, each practitioner was assigned to an image subject category for which they reported being deemed competent, whereas laypersons were evenly assigned across face, hand, and clothing image subject categories. All individuals who expressed interest in participating in the study were emailed a copy of *2022‐S‐0001: Standard Guide for Image Comparison Opinions* and a link to a Qualtrics survey. The first part of the Qualtrics survey was a participant questionnaire, in which additional information was collected, and the questions varied based on participant cohort. For instance, practitioners were asked about their last competency training, years of experience, and the number of comparisons they complete yearly, whereas laypersons were asked about the highest level of education received and exposure to forensic science (see Supporting Information 3 for a full list of questions asked to both practitioner and layperson participants). The second part of the Qualtrics survey involved participants assigning a source opinion category from *2022‐S‐0001* for each image pair sequentially. Through the online interface, the two images for a given pair were presented side‐by‐side with a click‐to‐zoom feature enabled only (images could not be downloaded). After viewing the two images, participants were required to select a source opinion category from *2022‐S‐0001* and answer some additional questions, including their self‐perceived difficulty of the comparison, confidence in their opinion, and time needed to complete the comparison (see Supporting Information 4 for the full list of questions). The order in which image pairs were presented was the same for all participants (i.e., no randomization). All individuals assigned to each image subject category (face, hands, or clothing) were given the same 20 pairs of images for comparison, regardless of whether they were a practitioner or layperson. Participants were not allowed to skip responses to any questions; the survey required participants to provide an answer to every question in order to proceed to the next. Participants were given 10 weeks to complete their questionnaire and comparisons through Qualtrics, after which point submissions were closed. Recruitment of participants, distribution of the survey via Qualtrics, and collection of information with informed consent were conducted with approval from the North Carolina State University Institutional Review Board (#26272).

### 2.3 Data validation and statistical analysis

Raw data was exported from Qualtrics and imported into Microsoft Excel (Version 16.89.1; Redmond, WA, USA) for quality control verification. All statistical analyses were performed using R (R version 4.4.2). Statistical analyses focused on four key areas: (1) The distribution of opinion source categories selected by participant cohorts across image subject categories was analyzed by first filtering the five *2022‐S‐0001* opinion levels—an ordinal scale defined as 5.1 (strong support for different sources), 5.2 (support for different sources), 5.3 (inconclusive), 5.4 (support for the common source), and 5.5 (strong support for the common source)—by cohort, image category, and image source. We then applied permutation‐based two‐sample Kolmogorov–Smirnov tests (10,000 permutations) to nonparametrically estimate *p*‐values for each comparison. (2) To assess reproducibility of opinion source categories across image subject categories, we first computed Cohen's kappa [15, 16]—weighted for ordinal data—for every unique rater pair within each cohort and for each image subject category (face, hands, clothing) [9]. For each image subject category, we then extracted the median *k* value for practitioners and for laypersons and then compared these medians between the two cohorts using two‐sided Wilcoxon rank‐sum tests (also known as the Mann–Whitney *U* test). (3) To assess the accuracy of opinion source categories selected by participant cohorts across image subject categories, we used the binary ground‐truth source information (same = 1, different = 0) provided in the University of Notre Dame metadata for all 60 pairs. Each participant's five‐point *2022‐S‐0001* opinion category ratings (5.1–5.5) were treated as an ordinal predictor, and a Receiver Operating Characteristic (ROC) curve was computed using the pROC package in R. The resulting area under the curve (AUC) values were calculated and served as each participant's accuracy score, with AUC = 1 denoting perfect accuracy [7, 8]. Practitioner and layperson AUCs were compared within each image category using two‐sided Wilcoxon rank‐sum tests. (4) The relationship between (a) participant‐rated difficulty (with the scale: “Easy” = 1, “Moderate” = 2, “Difficult” = 3, “Very Difficult” = 4, “Not Possible” = 5) and accuracy (AUC) and (b) self‐perceived confidence (with the scale: “Very Confident” = 1, “Fairly Confident” = 2, “Somewhat Confident” = 3, “Slightly Confident” = 4, “Not Confident at All” = 5) and accuracy (AUC) of selected opinion‐source categories was evaluated using Kendall's tau correlation [9] for both practitioner and layperson cohorts. We used a *p*‐value of 0.05 as the cutoff to claim statistical significance across all analyses.

## 3 RESULTS

### 3.1 Recruited participants

A total of 63 forensic practitioners and 100 laypersons registered their interest to participate in this study via the Google form. Each of these individuals was assigned an image subject category (face, hands, or clothing) either randomly (laypersons) or based on their reported competency (practitioners) and were sent the Qualtrics survey for completion. The completion rate—those who attempted and successfully completed the questionnaire and ≥45% of the assigned image comparisons in Qualtrics—was marginally higher for laypersons than for forensic practitioners (77% vs. 73%, respectively) (Table 1). This response rate was expected, given that changing time commitments or disinterest would lead to participant attrition in any survey. Forensic practitioner respondents (*n* = 38) represented 14 countries, mostly were employed by state/province or federal law enforcement agencies (74% combined), and the majority had been completing image comparisons for >2 years (87%). Based on our recruiting approach and the fact that there are more facial forensic examiners globally, it was not unexpected that over 80% of recruited practitioners were deemed competent in at least face comparisons. Layperson respondents (*n* = 44) represented five countries, and the overwhelming majority had graduated from college (89%; bachelor's, master's, and/or doctoral degrees). The breakdown of the laypersons into the three target subgroups was as follows: (1) 39% students (undergraduate or graduate) having completed forensic science coursework, (2) 36% individuals trained in forensic science but not in image comparisons, and (3) 23% general public with no formal exposure to forensic science. Notably, for subsequent data analyses, layperson participants were analyzed as a single cohort, given there was insufficient power to separate by subgroup and image subject category.

**TABLE 1:** Breakdown of the participants from both the forensic practitioner and layperson cohorts for the three image category types (face, hands, and clothing). (1) “Interested” participants were those who expressed interest via the Google Form and were sent the Qualtrics link to the survey; (2) “Attempted” participants were those who took any portion of the study (percentage calculated out of the number “interested”); and (3) “Fully completed” participants were those who completed (≥45% comparisons) the study (percentage calculated out of the number “attempted”).

	Practitioners			Laypersons		
	Interested	Attempted	Fully completed	Interested	Attempted	Fully completed
Face	30	25 (83%)	18 (72%)^a^	35	23 (66%)	16 (70%)^b^
Hands	17	11 (64%)	9 (82%)	34	21 (62%)	17 (81%)
Clothing	16	16 (100%)	11 (69%)	31	13 (42%)	11 (85%)
Total	63	52	38 (73%)	100	57	44 (77%)

^a^ Denotes that a single participant completed only 19 of the 20 assigned image comparisons.

^b^ Denotes that a single participant completed only 9 of the 20 assigned image comparisons.

### 3.2 Selection and distribution of opinion source categories

**FIGURE 3:**
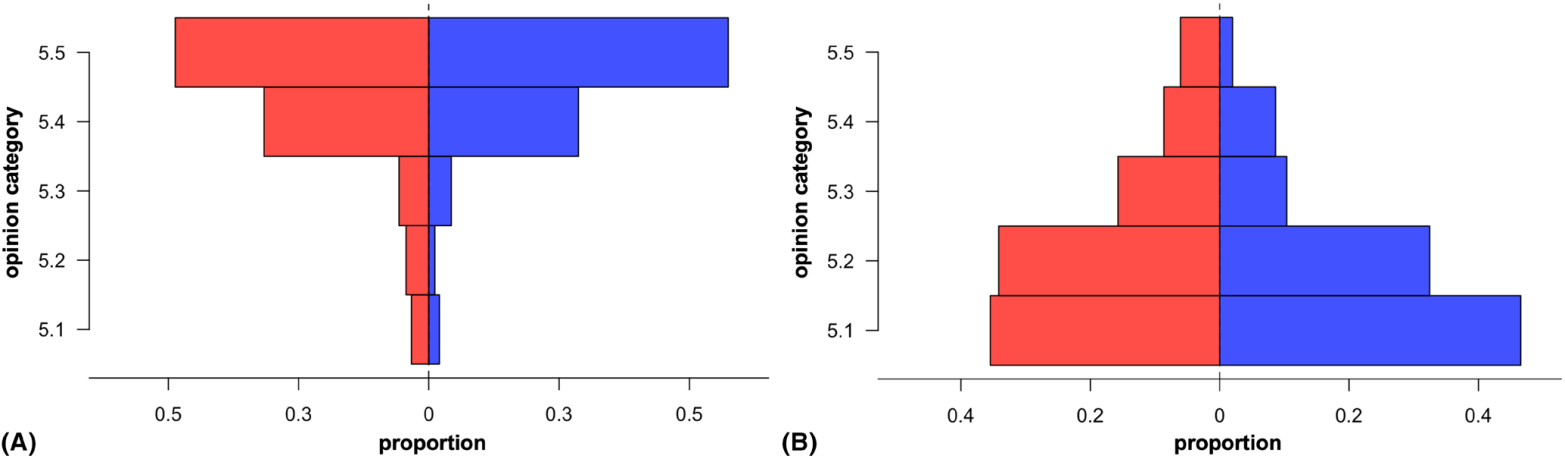
Selection of opinion categories from *2022‐S‐0001* (vertical axis) for common source identity image pairs (A) and different source identity image pairs (B) from the practitioner (blue; *n* = 38) and layperson (red, *n* = 44) participant cohorts.

In *2022‐S‐0001* five opinion categories are listed: 5.1—strong support for different sources, 5.2—support for different sources, 5.3—inconclusive, 5.4—support for the common source, and 5.5—strong support for the common source. Given that a previous facial comparison study observed a 7‐point identity scale being used differently depending on participant cohort and the image source [9], we examined whether similar trends would be observed in this study with more diverse image subject categories and participants. When comparing the use of the 5‐point scale in *2022‐S‐0001* between practitioners and laypersons, irrespective of image subject category or source, no statistical difference was observed between participant cohorts (*D* = 0.045, *p* = 0.395). A statistically significant difference in the selection of opinion categories between practitioners and laypersons was observed when examining only the common source (*D* = 0.094, *p* = 0.040) and only different source comparisons (*D* = 0.111, *p* = 0.021). This difference was visually reflected in histogram plots (Figure 3), where a higher proportion of practitioners selected the extremes of the scale (5.1 and 5.5) compared to laypersons. Notably, for different source comparisons (Figure 3B), both cohorts selected opinion categories somewhat more evenly across the full scale, whereas for common source comparisons (Figure 3A), over 85% of all participants selected either 5.4 or 5.5. These results indicate that participant cohorts used the 5‐point scale differently depending on image source type. Hahn and colleagues [9] also reported that opinion categories were used differently by different participant cohorts. In contrast to this study, in Hahn and colleagues [9], the practitioners (referred to as “examiners”) used all seven opinion categories more evenly, whereas super‐recognizers selected scale extremes. This contrasting result could be attributed to (a) the difference in the numbers of participants between studies—participants in each cohort were more even in this study (38 for practitioners and 44 for laypersons) when compared to Hahn and colleagues [9] (57 for examiners and 13 for super‐recognizers), or (b) the difficulty of the image comparisons presented to participants—Hahn and colleagues [9] used a three algorithm approach to select particularly challenging face image pairs for inclusion in their study (which was not the same approach used in this study).

Given that this study encompassed three different subject categories of images—face, hands, and clothing—we assessed whether the image subject impacted the selection of opinion categories for both participant cohorts. For the practitioners, a statistically significant difference was observed in the use of the 5‐point scale between hands and clothing (*D* = 0.168, *p* = 0.008) and between face and clothing (*D* = 0.183, *p* = 0.0002) image pairs (Supporting Information 5). For laypersons, a statistically significant difference was only observed between face and clothing (*D* = 0.127, *p* = 0.031) image pairs (Supporting INFORMATION 5). This result could in part have been attributed to the sub‐optimal quality of the clothing image pairs (i.e., clothing available for comparison limited to the neck and shoulders only). We additionally examined whether specific attributes and the experience of participants impacted how the opinion categories in *2022‐S‐0001* were used. No statistically significant differences were observed in the selection of opinion categories for the practitioner cohort based on (a) years of experience (<10 or >10 years; *D* = 0.0223, *p* = 1.00), (b) years since last competency training (<1 or >1 year; *D* = 0.0560, *p* = 0.620), (c) highest level of education received (bachelor's or below vs. advanced [masters and/or doctoral]; *D* = 0.047, *p* = 0.821), or (d) jurisdiction level of the employer (federal vs. state/province; *D* = 0.0347, *p* = 0.997). Similarly, no statistically significant differences were observed in the selection of opinion categories for the laypersons cohort based on (a) highest level of education achieved (bachelor's or below vs. advanced [masters and/or doctoral]; *D* = 0.046, *p* = 0.749) and (b) prior exposure to formal forensic science education and training (students vs. other forensic professional; *D* = 0.087, *p* = 0.185).

### 3.3 Agreement of the selected opinion categories between participants

One of the key goals of OSAC is to develop high‐quality, technically sound standards. In this study, we assessed the reproducibility of participants assigning a source opinion category for the same pair of images following *2022‐S‐0001*. All participants, regardless of cohort, assigned to a given image subject category (face, hands, or clothing) were given the same set of 20 image pairs for comparison. This design allowed us to assess the agreement between participants on the opinion category selected for each image pair. Agreement was assessed using Cohen's Kappa weighted for ordinal data, whereby values can range from +1 (indicating perfect agreement) to −1 (indicating complete disagreement) [17]. McHugh provides the following interpretation of Cohen's Kappa: >0.80 indicates strong agreement with 64%–81% of data being reliable; 0.60–0.79 indicates moderate agreement with 35%–63% of data being reliable; 0.40–0.59 indicates weak agreement with 15%–35% of data being reliable; and <0.39 indicates minimal to no agreement with <15% of data being reliable [17]. In this study, moderate agreement between practitioners on the selection of opinion source categories from *2022‐S‐0001* was observed for face and hands image pairs, whereas only weak agreement was observed for clothing image pairs (Table 2). Hahn and colleagues [9] also implemented Cohen's Kappa to assess agreement between practitioners for facial image comparisons and reported weak agreement (median 0.48). While the agreement between practitioners in this study for face image pairs was higher than that reported by Hahn and colleagues [9], there is one key difference between the studies: the larger number of participants (57 vs. 38 in this study). Several studies have reported that a larger sample size should lead to a more stable and reliable estimate of Cohen's Kappa, such that directly comparing the two studies with different sample sizes might not be scientifically sound. For practitioners, we also examined whether the level of agreement was impacted by (a) years of experience (<10 or >10 years), (b) highest level of education received (bachelor's or below vs. advanced [masters and/or doctoral]), and (c) years since last competency training (<1 or >1 year); none had a statistically significant impact. For laypersons, weak to minimal agreement between participants was observed for face and hands image pairs, whereas minimal agreement was observed for clothing image pairs (Table 2). We evaluated whether the level of agreement between practitioners was significantly different from the agreement between laypersons for each image category. As expected, we observed a higher level of agreement for practitioners over laypersons (Table 2), and the level of agreement was statistically different for face (*U* = 3889, *p* = <0.01), hands (*U* = 494, *p* = <0.01), and clothing (*U* = 739, *p* = <0.01) image pairs.

**TABLE 2:** Median agreement (Weighted Cohen's Kappa) between participants in the practitioner and layperson cohorts for selecting the same opinion category from *2022‐S‐0001* for each image pair. Results are presented separately per image subject category.

	Practitioners	Laypersons
Face	0.664	0.471
Hands	0.742	0.539
Clothing	0.548	0.362

### 3.4 Accuracy of participant cohorts in determining the opinion source

**FIGURE 4:**
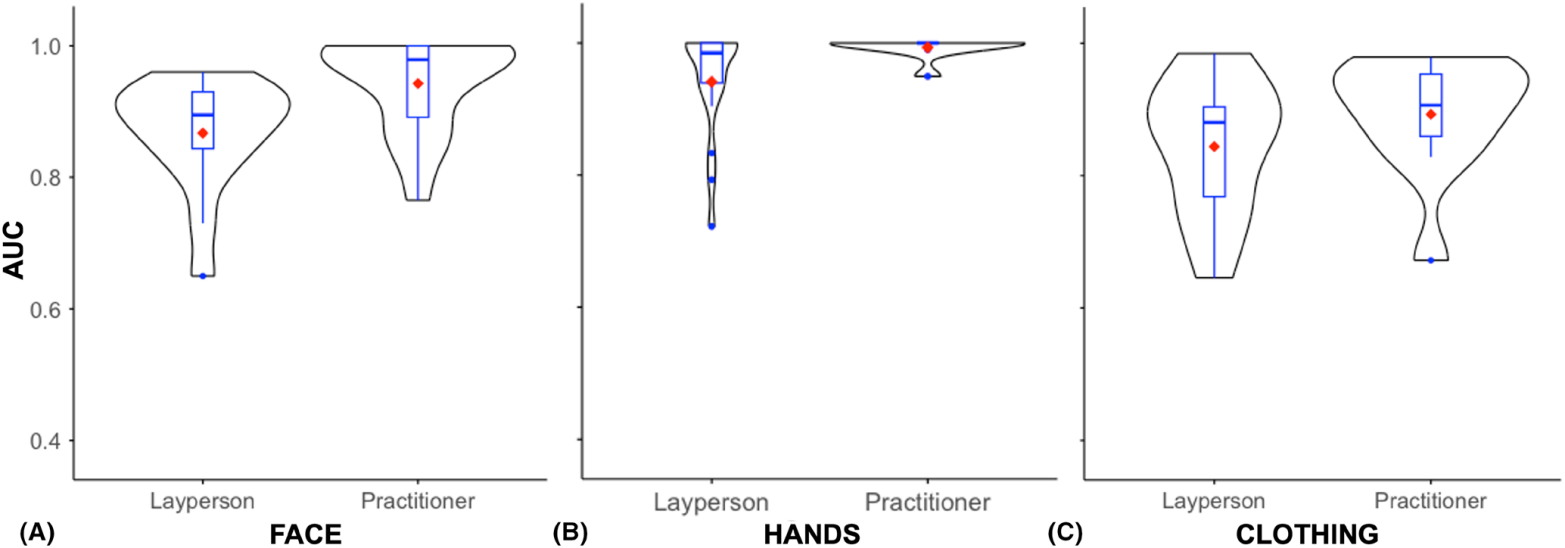
Distribution of accuracy (as measured by the area under the receiver operating characteristic curve [AUC]; vertical axis) for correctly selecting the true source (common or different) by each participant cohort for face (A), hands (B), and clothing (C) image pairs. The violin plot shows the range and probability density of AUC values, whereas the box‐and‐whisker plot overlay shows the median, interquartile ranges, and the full range of the AUC within each participant cohort. Points represent outliers. Red diamonds show the mean for each group.

The ground truth on the source (common or different) of each image in a pair was known for this study. Given this, we were able to assess the accuracy of participant cohorts for determining the source across face, hand, and clothing image subject categories. Figure 4 shows a violin plot with a box‐and‐whisker overlay of the AUC values for both participant cohorts and each image subject category, and Table 3 provides the mean AUC. For both face and hand image subject categories, a statistically higher AUC was observed for the practitioner cohort when compared to the layperson cohort (face: *U* = 61.5, *p* = 0.005; hands: *U* = 41.5, *p* = 0.043). No statistically significant difference in the AUC was observed between participant cohorts for clothing image pairs (*U* = 41.5, *p* = 0.224). Upon visual inspection of the distribution of AUC in Figure 4, it was evident that there was a larger range of AUC values across layperson participants for face and hand image pairs compared to the practitioner cohort. The increased accuracy of practitioners over laypersons in determining the image source for facial comparisons was previously reported by Phillips and colleagues [8], in which they highlighted that advanced training has a positive impact on accuracy.

**TABLE 3:** Mean accuracy (as measured by the area under the receiver operating characteristic curve [AUC]) between participants in the practitioner and layperson cohorts for selecting the truth source (common or different) for each image subject category.

	Practitioners		Laypersons	
	Mean	Range	Mean	Range
Face	0.943	0.765–1.000	0.867	0.650–0.960
Hands	0.993	0.949–1.000	0.941	0.722–1.000
Clothing	0.893	0.672–0.979	0.844	0.646–0.984

### 3.5 Correlation between self‐perceived difficulty/confidence and source accuracy

**FIGURE 5:**
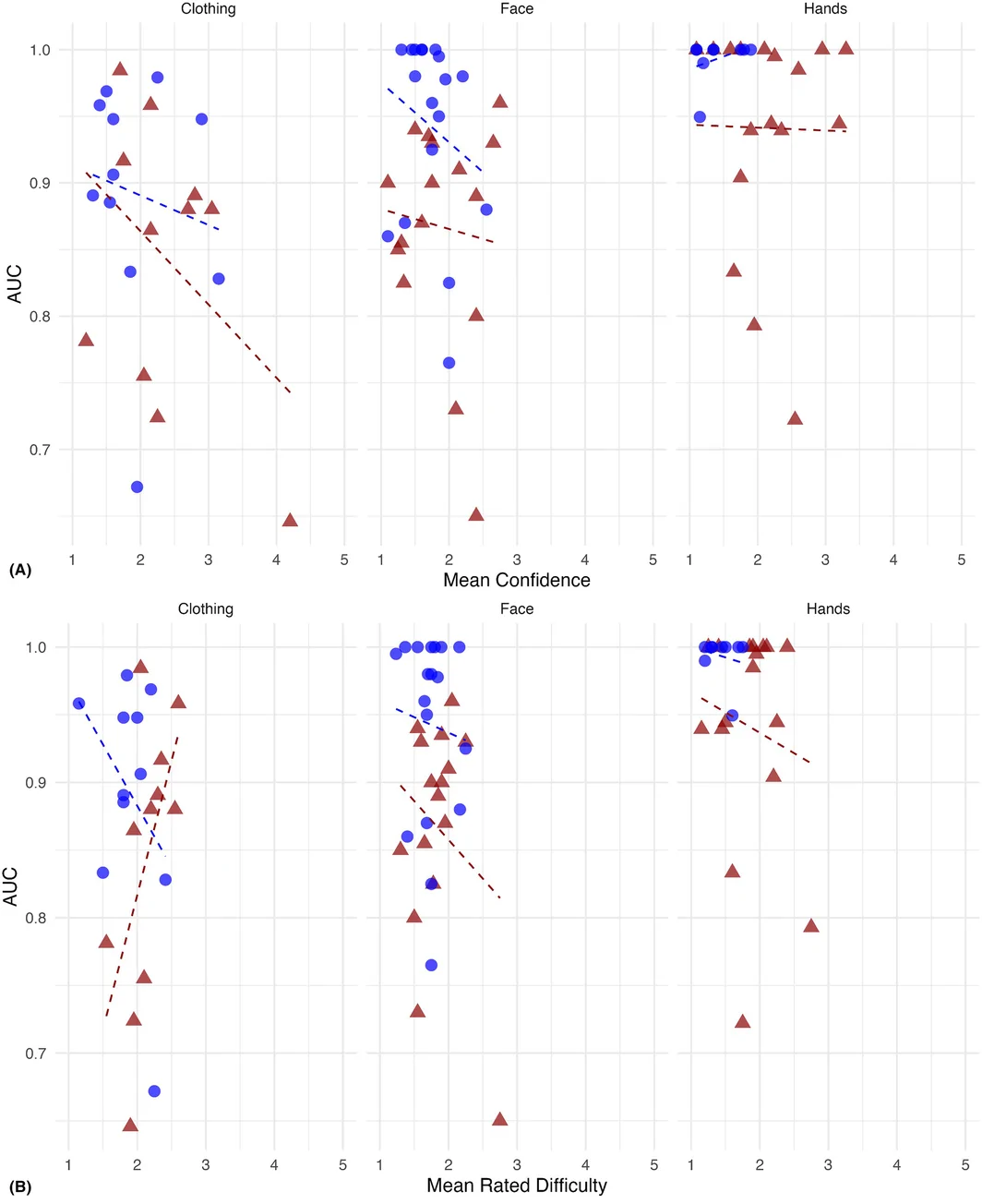
Scatter plots (with correlation trend lines) showing the relationship between self‐perceived confidence (A) and difficulty (B) on accuracy (area under the receiver operating characteristic curve, AUC) for clothing, face, and hand image pair comparisons. Blue dots/lines represent practitioners, whereas brown triangles/lines represent laypersons. Confidence scale: Very confident = 1, Fairly confident = 2, Somewhat confident = 3, Slightly confident = 4, Not confident at all = 5. Difficulty scale: Easy = 1, Moderate = 2, Difficult = 3, Very difficult = 4, Not possible = 5.

For every comparison, participants were asked to rate their self‐perceived: (a) difficulty (easy, moderate, difficult, very difficult, and not possible) and (b) confidence (very confident, fairly confident, somewhat confident, slightly confident, and not confident at all) in the opinion category they selected from *2022‐S‐0001* (Supporting Information 4). Capturing this information was important to gain insight into both the usability of *2022‐S‐0001* across diverse participant cohorts (i.e., if a participant struggled to understand either the instructions or the opinion categories described in *2022‐S‐0001*, they might have perceived the comparison as very difficult), but also to assess whether difficulty and confidence are linked to accuracy. To visually assess this, for each participant, their mean AUC across the 20 assigned image pairs was plotted against both their mean self‐perceived confidence and difficulty. We also assessed the relationship between both self‐perceived confidence and difficulty with AUC via Kendall's τ correlation. For the practitioner cohort, we observed a statistically significant correlation between accuracy and self‐perceived difficulty (*τ* = −0.259, *p* = 0.030) and between accuracy and self‐perceived confidence (*τ* = −0.234, *p* = 0.050). No statistically significant correlation between accuracy and either self‐perceived difficulty or confidence was observed for the layperson's cohort (self‐perceived difficulty: *τ* = 0.003, *p* = 0.976; self‐perceived confidence: *τ* = −0.022, *p* = 0.839). These results indicate that practitioners, likely due to their training and experience, have self‐awareness of their level of accuracy. Hahn and colleagues [9] also reported that practitioners and super‐recognizers had insight into their own accuracy for determining facial image pair source based on their self‐perceived difficulty and confidence. When assessing the relationship between both self‐perceived confidence (Figure 5A) and difficulty (Figure 5B) by participant cohort and image category type, in all situations, no significant differences were observed (Supporting Information 6). Notably, the small sample sizes available for these analyses might have impacted the ability to detect statistical differences.

## 4 DISCUSSION

In recent years, several scientific studies have been conducted to assess the accuracy and reproducibility of forensic image comparisons completed by trained practitioners (e.g., [7, 8, 9, 10, 11, 12, 13]). While each of these studies has provided valuable information on the accuracy and inferred error rates associated with forensic image comparisons, two main limitations among these studies exist: (1) the number of opinion categories (or scale) used for comparisons was not consistent between studies (e.g., 3, 5 or 7 categories), making it challenging to compare results between studies, and (2) no single study to date examined the performance of individuals in determining the image source for more than one image subject category. This global interlaboratory study looked to address these limitations by assessing the practical utility, accuracy, and reproducibility of assigning one of five opinion categories from the OSAC Proposed Standard *2022‐S‐0001* for pairs of face, hand, and clothing images with diverse participant cohorts.

While the results presented in this study add scientific value to the digital multimedia forensic disciplines, there are a few caveats to this study that may have impacted the reported results. Firstly, participants could not download the images from Qualtrics for import into the image software used in their laboratories for comparisons. Some practitioners noted that not being able to enhance key features of the images (via brightening, contrast, rotation, zoom, etc.) was a deviation from how they conduct image comparisons in casework and thus potentially impacted the opinion categories ultimately selected. While this was viewed as a study limitation by certain practitioners, this approach did provide a level of consistency in the delivery of image pairs (format, size, zoom, etc.) for all comparisons across each participant. Secondly, the study used an existing dataset of images. While variation was used as a factor in image selection, the images tended to be captured via a standardized method that is not typical of the more random or unconstrained nature of angles and/or poses seen in casework. Further, we did not have access to clothing‐specific images for use in this study; images from facial datasets where a large portion of clothing was visible were used. This approach meant that images included in the clothing subject category were not necessarily reflective of those encountered in forensic casework, especially as they rarely included clothing with visible signs of wear (tears, rips, etc.). This limitation might explain in part the lower agreement (Table 2) and lower accuracy (Table 3) for clothing comparisons when compared to face and hands. Thirdly, there were no time limits imposed on participants for completing the comparisons via Qualtrics. The survey was intentionally set up such that if a participant needed to complete the survey in multiple sittings, Qualtrics would save previous answers and allow them to resume completing the survey later without losing previously entered responses. The decision not to limit time for comparisons in this study was made with reference to previous studies that have examined the impact of varied exposure time on accuracy for facial comparisons [7]. Assessing whether the length of time available to complete a comparison impacted the use of opinion categories in *2022‐S‐0001* was outside the scope of this interlaboratory study but could be an avenue for future research. While outside the scope of this research, factors such as taking breaks between comparisons and additional workload outside of the study have the potential to impact results. Finally, it has been previously reported that facial practitioners who serve in a reviewer or assessor role use opinion categories (or scales) differently from practitioners who serve in an examiner role [8]. In this study, while we did collect information from facial practitioners as to their current role (examiner, reviewer, or assessor; Supporting Information 3), we did not have sufficient sample sizes to statistically assess whether the role impacted the use of opinion categories in *2022‐S‐0001*.

This global interlaboratory study aimed to assess the practical utility, accuracy, and reproducibility of OSAC Proposed Standard *2022‐S‐0001*. We highlighted that when individuals are provided with a clear and detailed framework of source opinion categories, moderate agreement and high accuracy (especially for practitioners) can be achieved. As expected, practitioners statistically outperformed laypersons when tasked with determining the truth source (common or different) for face and hand image pairs. A key pillar across all forensic science disciplines is consistency—ideally, two trained professionals given the same evidence would reach the same result. In this study, we examined consistency among practitioners and laypersons for selecting the same opinion category from *2022‐S‐0001* for a given pair of images. We reported higher agreement among practitioners for face, hand, and clothing image pairs over layperson participants, highlighting the importance of trained professionals when completing forensic image comparisons. One added benefit of this global interlaboratory study was that all participants were given the opportunity to provide feedback on *2022‐S‐0001*, specifically areas in which the language could be enhanced for clarity. These suggestions were compiled and forwarded to the Digital/Multimedia Scientific Area Committee (DM SAC) for consideration when preparing *2022‐S‐0001* for publication via a standards developing organization (SDO).

## FUNDING INFORMATION

This work was performed under the following financial assistance award 70NANB23H275 from the U.S. Department of Commerce, National Institute of Standards and Technology.

## CONFLICT OF INTEREST STATEMENT

The authors have no conflict of interest to report.

## ETHICS STATEMENT

Recruitment of participants and information collected were done so with informed consent, with North Carolina State University Internal Review Board approval (#24247).

## Supporting information


**Supporting Information 1.** Existing image datasets maintained by the University of Notre Dame Computer Vision Research Laboratory and the National Institute of Standards and Technology (NIST) that were licensed to North Carolina State University for use in this study.

**Supporting Information 2.** Technical details for machine learning and computer vision algorithms for image prefiltering.

**Supporting Information 3.** Participant questionnaire administered via Qualtrics to both forensic practitioners and laypersons.

**Supporting Information 4.** List of questions administered to both forensic practitioners and layperson participants via Qualtrics for each of the 20 image pairs.

**Supporting Information 5.** Selection of opinion categories from *2022‐S‐0001* (vertical axis) by practitioners (*n* = 44) for hands and clothing image pairs (A), by practitioners (*n* = 44) for face and clothing image pairs (B), and by laypersons (*n* = 38) for clothing and face image pairs (C).

**Supporting Information 6.** Results from Kendall's tau to assess correlation between (a) self‐perceived confidence (with the scale: “Very Confident” = 1, “Fairly Confident” = 2, “Somewhat Confident” = 3, “Slightly Confident” = 4, “Not Confident at All” = 5) and accuracy (AUC) and (b) participant‐rated difficulty (with the scale: “Easy” = 1, “Moderate” = 2, “Difficult” = 3, “Very Difficult” = 4, “Not Possible” = 5) and accuracy (AUC) of selected opinion‐source categories. Results are broken down by participant cohort and image category type. * denotes the adjusted *p*‐value is reported.

## Data Availability

The data that supports the findings of this study are available in the Supporting Information of this article. Additionally, the automation code, the in‐house hand image selection algorithm, and the detailed documentation is available at https://bitbucket.org/leecwwong/image‐comparison/.
