# Supplementary material for: Global interlaboratory study: Assessing consistency and accuracy for determining source of face, hands, and clothing images across forensic practitioners and laypersons
Source: J Forensic Sci. 2026 Jun 15;71(5):2088–98. doi: 10.1111/1556-4029.70368 (PMC13534925; doi:10.1111/1556-4029.70368)
Supplement: Supplementary file 1 [file JFO-71-2088-s001.docx]

**SUPPORTING INFORMATION 2.** Technical details of machine learning algorithms for image prefiltering

We used computer algorithms to preselect 100 pairs of face images, 100 pairs of hand images, and 45 pairs of clothing images from approximately 125,000 images licensed from the University of Notre Dame Computer Vision Research Laboratory and the National Institute of Standards and Technology (NIST) (see **SUPPORTING INFORMATION 1** for details of the datasets). The prefiltering algorithms for face and clothing images were built on open-source software, and the prefiltering algorithm for hand images was developed in-house due to the lack of publicly available software. We detailed the image prefiltering processes for face, clothing, and hand images below, respectively.

Our prefiltering algorithms and detailed documentation are publicly available at https://bitbucket.org/leecwwong/ image-comparison/.

*2.1. Face image selection*

100 selected pairs of face images fall into one of the four categories, with 25 pairs in each category:

- Same source - easy comparison
- Same source - difficult comparison
- Different source - easy comparison
- Different source - difficult comparison

Every 25-pair category consisted of 5 pairs from each of the 5 datasets in the following table:

| **Dataset** | **# of Available Images** |
| --- | --- |
| HID-data-B | 11,004 |
| ND Female Male Accuracy | 5,515 |
| Twins 2009-2010 | 2,713 |
| BVC-UNN_Face Data Set | 2,333 |
| Point&Shoot Challenge | 2,236 |

The number of available images from each of the above datasets is smaller than that in the licensed dataset. We used SynergyNet^[1]^ (Wu et al., 2021) to estimate the facial orientation and excluded images with non-frontal views (*i.e.,* the sum of the absolute values of yaw, pitch, and roll > 5°). We did not use other datasets licensed as in **SUPPORTING INFORMATION 1** since they had too few photos once the frontal-view constraint was applied. Excluding datasets with few images reduces the likelihood of our participants seeing images used in other studies.

We measured the pairwise similarity of any two face images using the cosine distance between their embedding vectors, each generated from applying the DeepFace^[2]^ (with the VGG-Face + MtCnn option) neural network to a face image. We oversampled more candidate image pairs than 100 pairs to allow for manual filtering away of unqualified image pairs. We removed images that were blurry, underexposed, with significant facial expressions, and shadows on the face. We tried to avoid constructing pairs that are trivial. For example, our same-source easy pairs excluded photos taken on the same day, whereas different-source difficult pairs excluded pairs with a man and a female, a small age gap, and similar skin color/race.

*2.2. Clothing image selection*

45 selected pairs of face images fell into one of the two categories:

- Same source (25 pairs)
- Different source (20 pairs)

Clothing images were selected from the same 5 datasets as for the face images, namely, HID-data-B, ND Female Male Accuracy, Twins 2009-2010, BVC-UNN_Face Data Set, and Point&Shoot Challenge. For the same-source images, 5 image pairs were selected from each of the 5 datasets.

For the different-source images, 5 image pairs were selected from each of the 4 datasets, excluding the Twins 2009-2010 dataset, in which twins wore the same clothing when taking photos.

We measured the pairwise similarity of two clothing images using the cosine distance between the embedding vectors, each generated from applying the market1501_resnet50_256_128_epoch_120 checkpoint of centroids-reid neural network^[3]^ (Wieczorek et al., 2021) to an image.

*2.3. Hand image selection*

80 selected pairs of hand images that fell into one of the four categories, with 20 pairs in each category:

- Same source - easy comparison
- Same source - difficult comparison
- Different source - easy comparison
- Different source - difficult comparison

We used images from the HID-hand dataset only to select the 80 pairs of hand images.

To construct the same-source image pairs, we used the image acquisition time available from the metadata of the HID-hand dataset to determine the level of difficulty. Easy pairs were constructed from images collected on the same day, whereas difficult pairs (of the same person) were collected on different days.

To construct the different-source image pairs, we evaluated the visual similarity of ~149,000 hand image pairs and picked the 20 most similar pairs and designated them as “difficult” and the 20 least similar pairs as “easy”. The selected pairs were visually validated by the computer vision expert on the author team to ensure that the difficult pairs (from different persons) are indeed more difficult than the easy pairs.

The visual similarity for each pair of different-source images was quantified using our in-house similarity measure algorithm, as other similarity algorithms in the biometrics literature (Cheng & Kumar, 2020; Li & Zhang, 2021; Cheng & Kumar, 2021; Zhou & Kumar, 2024) were not publicly available. The high-level idea of our in-house algorithm is as follows. First, a deep-learning-based pixel-level motion estimation and compensation algorithm (Jiang et al., 2021) was applied to warp the first hand image to resemble the second hand image, so that the shape from the first hand image aligns with that in the second image. Second, with the first hand image slightly distorted but aligned to the second hand image, we were able to quantify its visual similarity with respect to the second image via three widely used pixel-level similarity metrics (*i.e.,* SSIM [Wang et al., 2004], multi-SSIM [Wang et al., 2003], and PSNR [Gonzalez & Woods, 2018]).

We acknowledge that while our in-house algorithm for hand similarity measure does not explicitly measure factors such as knuckle patterns, vein patterns, and hand shape as in the biometrics literature, it implicitly captures the dissimilarity in knuckle patterns and vein patterns. We note that the application scenario of our motion-estimation-based hand similarity measure should be limited to the image prefiltering tasks pending further downselection by subject matter experts.

***References***

Wu CY, Xu Q, Neumann U. Synergy between 3DMM and 3D landmarks for accurate 3D facial geometry. In: IEEE International Conference on 3D Vision (3DV); 2021. pp. 453–463.

Wieczorek M, Rychalska B, Dąbrowski J. On the unreasonable effectiveness of centroids in image retrieval. In: Mantoro T, Lee M, Ayu MA, Wong KW, Hidayanto AN, editors. Neural Information Processing—ICONIP 2021. Lecture Notes in Computer Science. Cham: Springer; 2021. Vol. 13111.

Cheng KH, Kumar A. Contactless biometric identification using 3D finger knuckle patterns. IEEE Trans Pattern Anal Mach Intell. 2019;42(8):1868–1883.

Li S, Zhang B. Joint discriminative sparse coding for robust hand-based multimodal recognition. IEEE Trans Inf Forensics Security. 2021;16:3186–3198.

Cheng KH, Kumar A. Deep feature collaboration for challenging 3D finger knuckle identification. IEEE Trans Inf Forensics Security. 2020;16:1158–1173.

Zhou Z, Kumar A. Finger-knuckle assisted slap fingerprint identification system for higher security and convenience. IEEE Trans Inf Forensics Security. 2023;19:441–454.

Jiang S, Campbell D, Lu Y, Li H, Hartley R. Learning to estimate hidden motions with global motion aggregation. In: IEEE/CVF International Conference on Computer Vision (ICCV); 2021. pp. 9772–9781.

Wang Z, Simoncelli EP, Bovik AC. Multiscale structural similarity for image quality assessment. In: The Thirty-Seventh Asilomar Conference on Signals, Systems & Computers; 2003. pp. 1398–1402. Pacific Grove, CA, USA: IEEE.

Zhou W, Bovik AC, Sheikh HR, Simoncelli EP. Image quality assessment: From error visibility to structural similarity. IEEE Trans Image Process. 2004;13(4):600–612.

Gonzalez RC, Woods RE. Digital Image Processing. 4th ed. Upper Saddle River, NJ: Pearson Education; 2018.

1. Ranked the second place in the head pose estimation task on the AFLW2000 dataset as of Jan. 16^th^, 2024 on the Papers With Code leaderboard. Source code available at https://github.com/choyingw/SynergyNet
2. Source code available at https://github.com/serengil/deepface
3. Source code available at https://github.com/mikwieczorek/centroids-reid
