# Supplementary material for: Global interlaboratory study: Assessing consistency and accuracy for determining source of face, hands, and clothing images across forensic practitioners and laypersons
Source: J Forensic Sci. 2026 Jun 15;71(5):2088–98. doi: 10.1111/1556-4029.70368 (PMC13534925; doi:10.1111/1556-4029.70368)
Supplement: Supplementary file 2 [file JFO-71-2088-s002.docx]

**SUPPORTING INFORMATION 4.**

***Questionnaire – opinion category assignment***

***[The same set of questions was asked for each of the 20 image pairs]***

- What is the unique code assigned to this set of images?
- Which of the following opinion categories from 2022-S-0001 would you select for this pair of images?
  - 5.1 Strong support for different source
  - 5.2 Support for different source
  - 5.3 Inconclusive
  - 5.4 Support for common source
  - 5.5 Strong support for common source
- Using the opinion scale you currently use in casework, what opinion would you conclude for this pair of images?
- Please rate the difficulty of the image comparison using a previously published 5-point difficulty rating scale.
  - Easy: The comparison was easier than most image comparisons
  - Moderate: The comparison was a typical image comparison
  - Difficult: The comparison was more difficult than most image comparisons
  - Very Difficult: The comparison was unusually difficult, involving significant photometric illumination, or pose changes, other red flags
  - Not possible: The comparison was virtually impossible, due to a lack of detail in the image(s)
- How confident are you in the opinion reached?
  - Not confident at all
  - Slightly confident
  - Somewhat confident
  - Fairly confident
  - Very confident
- Were there any notable characteristics that helped you reach the opinion?
- Please estimate the length of time needed to complete the comparison
  - <5 minutes
  - 5-9 minutes
  - 10-29 minutes
  - 30-59 minutes
  - 1-2 hours
  - >2 hours
- Please provide details as to whether you processed the images (e.g., changed contrast, removed color, etc.) to assist with the comparison
