# Supplementary material for: Global interlaboratory study: Assessing consistency and accuracy for determining source of face, hands, and clothing images across forensic practitioners and laypersons
Source: J Forensic Sci. 2026 Jun 15;71(5):2088–98. doi: 10.1111/1556-4029.70368 (PMC13534925; doi:10.1111/1556-4029.70368)
Supplement: Supplementary file 4 [file JFO-71-2088-s004.docx]

**SUPPORTING INFORMATION 3.**

***Questionnaire – participant information***

**PRACTITIONERS**:

- Please provide the name of your employer.
- What is the jurisdiction level of your employer? (Please select all that apply)
  - Global
  - Federal
  - State/Province
  - Local
  - Private
  - Other, please describe
- What country is your employer located in?
- What is the highest level of education you have received?
  - Middle school
  - High school
  - Some college
  - Associate degree
  - Bachelor’s degree
  - Master’s degree
  - Doctoral degree
  - Other, please describe
- What was your sex assigned at birth?
  - Male
  - Female
  - Prefer not to disclose
- What is your age?
  - 18-24
  - 25-34
  - 35-44
  - 45-54
  - 55-64
  - 65 and over
  - Prefer not to disclose
- When did you last complete competency training?
  - <1 year
  - 1-2 years
  - 2-5 years
  - 5-10 years
  - >10 years
- Who was the competency training administered by?
- Which image categories were you deemed competent in?
  - Faces
  - Irises
  - Clothing
  - Hands
  - Vehicles
  - Buildings
  - Fingerprints
  - Friction ridge
  - Feet
  - Other, please describe
- How many cases do you complete on average per year?
- How many comparisons do you complete on average per year?
- What is your position title?
- How long have you been conducting image comparisons?
  - <1 year
  - 1-2 years
  - 2-5 years
  - 5-10 years
  - >10 years
- Which of these roles do you fall into?
  - Reviewer
  - Assessor
  - Examiner
- Were there any areas of the standard that were ambiguous or unclear to you? If so, please provide details.
- Please briefly describe the opinion scale used by your employer.

**LAYPERSONS**

- What is your involvement in forensic science? (Please select all that apply)
  - Work in a forensic laboratory but not in image comparison
  - Completed (or currently completing) graduate training in forensic science
  - Completed (or currently completing) undergraduate training in forensic science
  - No involvement in forensic science
  - Other, please describe
- Have you received or participated in any trainings/coursework related to forensic image comparisons?
  - Yes
  - No
- Which country do you reside?
- What is the highest level of education you have received?
  - Middle school
  - High school
  - Some college
  - Associate degree
  - Bachelor’s degree
  - Master’s degree
  - Doctoral degree
  - Other, please describe
- What is your sex assigned at birth?
  - Male
  - Female
  - Prefer not to disclose
- What is your age?
  - 18-24
  - 25-34
  - 35-44
  - 45-54
  - 55-64
  - 65 and over
  - Prefer not to disclose
- Were there any areas of the standard that were ambiguous or unclear to you? If so, please provide details.
