# Supplementary material for: Global interlaboratory study: Assessing consistency and accuracy for determining source of face, hands, and clothing images across forensic practitioners and laypersons
Source: J Forensic Sci. 2026 Jun 15;71(5):2088–98. doi: 10.1111/1556-4029.70368 (PMC13534925; doi:10.1111/1556-4029.70368)
Supplement: Supplementary file 5 [file JFO-71-2088-s006.docx]

|  | | ***AUC vs. Confidence*** | | ***AUC vs. Difficulty*** | |
| --- | --- | --- | --- | --- | --- |
|  |  | **τ** | **p-value*** | **τ** | **p-value*** |
| ***Practitioners*** | ***Face*** (n = 18) | -0.260 | 0.523 | -0.014 | 0.966 |
|  | ***Hands*** (n = 9) | 0.310 | 0.523 | 0.089 | 0.966 |
|  | ***Clothing*** (n = 11) | -0.220 | 0.523 | -0.112 | 0.966 |
| ***Laypersons*** | ***Face*** (n = 16) | 0.051 | 0.786 | 0.202 | 0.837 |
|  | ***Hands*** (n = 17) | -0.073 | 0.786 | -0.008 | 0.966 |
|  | ***Clothing*** (n = 11) | -0.241 | 0.523 | 0.537 | 0.138 |

**SUPPORTING INFORMATION 6.**
