# Supplementary material for: Global interlaboratory study: Assessing consistency and accuracy for determining source of face, hands, and clothing images across forensic practitioners and laypersons
Source: J Forensic Sci. 2026 Jun 15;71(5):2088–98. doi: 10.1111/1556-4029.70368 (PMC13534925; doi:10.1111/1556-4029.70368)
Supplement: Supplementary file 6 [file JFO-71-2088-s003.docx]

**SUPPORTING INFORMATION 5.**
